# “The Mole on His Penis Lassos Her:” Cultural Understandings of Coercive Control and Emotional Abuse of Women in Cambodia

**DOI:** 10.1177/10778012231174349

**Published:** 2023-06-12

**Authors:** Maurice Eisenbruch

**Affiliations:** 1 Department of Psychiatry, School of Clinical Sciences at Monash Health, 2541 Monash University, Clayton, Victoria, Australia; 2 Royal University of Phnom Penh, Phnom Penh, Cambodia

**Keywords:** birthmarks, coercive control, gender-based violence, impunity, omens

## Abstract

Throughout world history, moles and birthmarks have occupied a special place as omens. Little is known of the cultural beliefs concerning the determinants of coercive control. In this ethnographic study of coercive control in Cambodia, the focus is on popular beliefs that moles are omens portending that men shall control women. Lachrymal moles (under the eye) signify women weeping as a result of misery. Penile moles portend men attracting, controlling, even abusing women. They have implications for reinterpreting an “insider” view of hegemonic masculinity and for culturally responsive interventions against gender-based violence.

Throughout history, moles and birthmarks have occupied a special place as omens. The legendary Greek soothsayer Melampous inspected moles for their prophetic and psychological meaning, using Mesopotamian divination: from head to heels; on the right and the left; and in lists of conditional sentences with *protasis* and *apodosis*, that if the mole is located on a particular location, such-and-such a thing will befall the person (Dasen, 2015). This is relevant to Cambodian divination, given its origins in Vedic India, which, in turn, draws from Mesopotamian divination.

Dasen noted that moles have been thought of as intensifiers of anatomical functions, such as sexual voracity when found in a “hidden place,” or *krypton* (p. 156).” Jaberg (1957) discussed Thomas Carew's (1595–1640) *Upon a Mole in Celia's Bosom*, where Hybla is a Sicilian mountain that is abundant in thyme, to which bees are attracted. Celia's breasts are likened to a pair of Hyblas, where the bee nests and flies to feast on the dew in the valley between them, only to drown in it, and its shadow remains a mole on Celia's breast. All those who are attracted by it will experience both the “sweetness of the honey and the anguish of the sting (p. 328).”

## Coercive Control

A central concern is the cultural framing of coercive control (McKinley & Liddell, 2022; Sharp, 2014; Stark, 2009) and the signs that particular men and women are destined to become embroiled in coercive relationships, but, although there are cross-cultural reports as well studies on the structural factors in social entrapment and coercive control (Obeid et al., 2010; Stark & Hester, 2018; Thananowan & Heidrich, 2008), there is a dearth of ethnographic literature on the “insider view” of coercive control. Intimate partner violence (IPV) is a serious issue in Cambodia (Ang & Lai, 2022; Lilja & Baaz, 2015; Mai & Phyu, 2020; Miedema et al., 2022), and various authors have flagged masculinity (Diakonia, n.d.; GADC, 2010), sexual coercion (Farvid & Saing, 2021) and coercive control (Brickell, 2020; Fulu et al., 2015; GADC, 2010; Miedema et al., 2022), as a feature of many intimate relationships in Cambodia. Judging by the above authors, it seems to have stayed that way. During the Khmer Rouge regime, the hollow claim was made that women were “liberated” from gender inequality (Tkachenko, 2019).

Acceptance of IPV is said to be entrenched in cultural norms that are reinforced in literary texts and proverbs, most notably, the classical “Code of Women's Behavior,” a didactic text of a mother offering instruction to her daughter on proper behavior as both a woman and wife. The text is said to have been written in the early 19th century as part of a misogynist element emerging in Cambodian elite society (Jacobsen, 2012), which prescribes a women's submissive position (Aveling, 2012; Derks, 2008; Surtees, 2003) in the family and society. The cultural message of this Code is one that projects concerning women's empowerment strive to overcome (Jacobsen, 2008). Even today, according to Chau (2017), with the removal of the Code from the school curriculum, which continues to influence society's view of women and the rates of IPV.

## Moles in Cambodia

While the Code is undoubtedly important, in this article, I argue for a more nuanced understanding of the cultural epigenesis of gender-based violence (GBV) and IPV. I examine the subtle forms in which coercive control is exercised, specifically through cultural beliefs around magical forms of control, namely moles /prɑcruy/ 
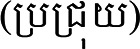
, which are no small matter in traditional Cambodian society, given the belief that a birthmark, a residue from one's previous life (Prigent, 2015), reveals information on a child's karmic past and destiny (Bennett, 2015; Davis, 2015; Lenart et al., 1991; Wekemann, 2016).

This study of moles arose from my ethnographic research on the cultural context of IPV in Cambodia. I have examined the manner in which moles form part of the “cloak of impunity” (Eisenbruch, 2018a), the astrological context of moles in risk perception and vulnerability to mishap and disaster (Eisenbruch, 2020), and the belief that moles are markers of the karmic rebirth of boys who are destined to perpetrate violence in the next generation, with all this being part of the “cultural epigenesis” of IPV (Eisenbruch, 2018b, 2019). I propose that another way of looking at this is that moles could be considered an indicator of what, in western terms, could be framed as coercive control, in rural Cambodia. In a study of the emotional bond between intimate partners, some perpetrators blamed the moles on their penises for drawing vulnerable women to them (Eisenbruch, 2018a, 2018b). In a second incident involving “love triangles” that led to an acid attack by a jealous partner, the penile mole was seen as setting up a toxic triangle and destroying a marriage. In a third incident involving Buddhist monks and child sexual abuse (CSA), it was surprising to find cases where monks blamed their penile moles for seducing young girls. I found that moles in particular locations, such as the face, breasts, hands, and genitals, were thought to predict national disaster. This was the starting point for this study. Addressing audiences in Cambodia on the topic of moles—penile moles in particular—as omens of emotional, physical, and/or sexual violence sometimes evoked strong reactions. Cambodians were enlivened that a westerner was focusing on a topic that many knew about but few felt they had been given the opportunity to discuss with western audiences, whereas one western-funded Non-governmental organization (NGO) misinterpreted my presentation on the penile mole as advocating for IPV—an illustration how this kind of research may lead to conclusions that are not as intended but rather framed within a specific western mindset that does not allow for alternative explanations. The preliminary findings on the penile moles suggest that there is an undeniable cultural idiom that may serve as a window into the manner in which coercive control works on ground. I argue that culturally responsive programs to stop IPV or CSA must have, as a starting point, evidence on the focus on the views of perpetrators, survivors, families, communities, lay people, and Buddhist monks and officiants, on the significance of omens, including genital moles.

## Roadmap

In this article, I consider the cultural significance of moles. I start with the image of the penis as a “weapon of power” in coercive sex in societies where there is male hegemony. I examine the manner in which “magic” and “poisonous enchantment” add to the equation. I consider the literature from India and Southeast Asia, on birthmarks as omens of misfortune and how they are thought to appear in cycles of rebirth in karmic societies. The theoretical underpinning of moles as distal omens of coercive control is “embodied uncertainty,” which I have examined in risk perception and vulnerability to mishap in Cambodia (Eisenbruch, 2020). I show that moles, as omens, are far from being simple “if this, then that” conditional statements that link the presence of a mole with a given consequence. Rather, they are statements that both communicate social realities in a community and work as graphic examples of how embodied uncertainty works in the production of omens of mishap. I argue that moles are considered good or bad omens and that the interpretation of an omen depends on the location of the mark.

After presenting material from the traditional healing manuals showing the topography of being “blighted,” I discuss six case studies. In the first two, I discuss the lachrymal mole that is the “scaffold of tears.” The case of Ly Sophoan shows how her mole was a mark of her karmic destiny to suffer tears of grief throughout her life cycle. The case of Hanni shows the role of inheritance from a previous life that was believed to be connected to the rape of a child. I then discuss the “hidden mole” on the genitals, in particular, the penis. The case of Rithy is a typical story of a man whose penile mole was considered responsible for drawing in dozens of vulnerable women. It shows how the mole ultimately wrecked both his life and the lives of the women he was involved with. The case study of Soriya shows the pernicious consequences in which a man in a “love triangle,” said to have lost his judgment as a result of his penile mole, ended up facing an acid attack at the hands of his enraged wife. The case study of Buntha is the story of a chief monk who raped and impregnated a child, and blamed his conduct on a mole, and shows that Buddhist monks, despite their monastic code of conduct, are ordinary men and are not exempt from the proclivities imposed by a genital mole. I then discuss moles in other locations. The case study of Rachada shows how a mole on her tongue was believed to lead to the death of her husband. Finally, I consider some of the popular beliefs in present-day Cambodia as reflected in social media.

## Methods

The research questions were framed in consultation with community members, including Buddhist monks and those who had lived experiences of coercive control (Tajima, 2021).

### Procedure

As part of our Aṅgulimāla Walks program, we obtained ethics approval from the National Ethics Committee for Health Research in Cambodia and from Monash University in Australia. We used our existing networks of village leaders, Buddhist monks, and lay ritual officiants to identify suitable families. The Sexual Violence Research Initiative's Ethical Guidelines state that oral consent is preferable (Jewkes et al., 2012). Based on the Participant Explanatory Statement and our nuanced prompts, we endeavored that our research would not be misconstrued as condoning IPV as a cultural or religious aspect of Cambodian life. Informants’ names and locations have been anonymized. The fieldwork encounters were carried out between 2014 and 2022 by the author, a male medical anthropologist and transcultural psychiatrist based in Australia, and two Cambodian assistants (Mr CA and Ms CP). In terms of researcher positionality (Tajima, 2021), the author writes as a middle-class man with an acquired understanding of Cambodian Buddhism and the Khmer language, but with sufficient cultural humility to appreciate the expression, “Khmer speech, foreign heart.” Mr CA is a male native Cambodian. He has trained under me in the ethnographic method and has engaged in fieldwork with me in urban and rural areas, for over 30 years now. He has longstanding relations with many of the informants. Ms CP is a female native Cambodian with a background in Khmer linguistics who had worked with the author for 12 years both in Australia and Cambodia at the time of writing. Other female and male research assistants were recruited from time to time. Wherever possible, women interviewed women, and men interviewed men. However, this became difficult between 2020 and late 2022, owing to COVID-19-related restrictions, following which the author and Ms CP traveled from Australia to Cambodia. The data for this study came from a series of fieldwork projects conducted in Phnom Penh, Kampong Speu, Prey Veng, Pursat, Kandal, Ta Keo, Svay Rieng, Kampong Chhnang, Banteay Meanchey, and Siem Reap provinces. Informants were gathered by snowball sampling. The initial informants came from the fieldwork on IPV. One woman felt ineluctably drawn to enter a relationship with a man who had a reputation as a womanizer. A second woman felt trapped in a marriage and felt that her life would be better off if she left her husband but found herself inexplicably incapable of doing so. A third man had a reputation of being a womanizer and revealed that he had a penile mole. We included these cases, and met their partners and other family members, as well as key informants such as Buddhist monks from whom the informants had sought help.

We documented the informants’ views on moles, particularly penile, /prɑcruy lɨŋ/ 
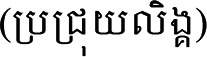
 and sublachrymal moles, literally, “mole that is the scaffolding for tears,” /prɑcruy rɔɔŋ tɨk pnɛɛk/ 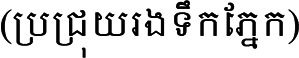. We explored the beliefs around their presumed predictive power as omens, for example, a “mole of the incapacity to have good fortune,” /prɑcruy ʔaʔpʰoap/ 
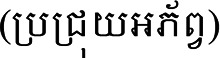
, and their effects on their relationship with their intimate partners or on those of family members and their partners. We recorded their use of language, proverbs, sayings, and their views on relevant folklore. All fieldwork was conducted in Khmer.

### Analysis

The author and CP relied on grounded theory and conducted a modified thematic analysis to seek the insider views of people within the culture and a narrative inquiry of the storied lives of particular case studies (Bhattacharya, 2017; Levitt et al., 2018; Pham et al., 2021). The starting point was a “theoretical sensitivity” (Thistoll et al., 2016) acquired through years of fieldwork experience with CSA in Cambodia. We started by identifying the description of the moles on men and women, their locations on the body, and the presumed effects of their presence in these spots. We analyzed the metaphors conveyed by the informants, for example, the depiction of the action as “lassoing” or “weeping,” to supplement the meanings attributed to the moles.

Table 1 shows the placement of inauspicious moles among those surveyed. IPV is of four kinds (coercive control and emotional violence with the “lassoed” partner, acid attack, abuse by monks and occasionally by ritual officiants, and suicide or attempted suicide in the context of a conflictual relationship). Each subcategory is divided into three, based on the location of the mole on the male or female genitals, under the eyes, or the breast, chin, or throat. The next two columns show whether the mole was located on the body of the perpetrator/the controlling partner/the abuser versus on the body of the person who suffered and was abused. The final column shows the number of males and females.

**Table 1.** Placement of Inauspicious Moles.

Category	Context	Type of mole	Mole on perpetrator, controlling partner, or abuser	Mole on victim or the abused person	Fieldwork by sex
IPV	Acid attack	Genital	*	*	*F* = 2, *M* = 7
		Lachrymal			*F* = 0, *M* = 0
		Other (*breast)		*	*F* = 4, *M* = 0
	Coercive control and emotional violence with partner	Genital	*		*F* = 3, *M* = 12
		Lachrymal		*	*F* = 5, *M* = 3
		Other—nostril, chest		*	*F* = 1, *M* = 1
	Monk (and acaa) abuse	Genital	*		*F* = 0, *M* = 1
		Lachrymal			*F* = 0, *M* = 0
		Other			*F* = 0, *M* = 0
	Suicide (complete or attempted) in the context of IPV	Genital		*	*F* = 0, *M* = 2
		Lachrymal			*F* = 1, *M* = 0
		Other			*F* = 0, *M* = 0
	Subtotal:				Genital: *F* = 0, *M* = 22 Lachrymal: *F* = 8, *M* = 3 Other: *F* = 5, *M* = 1
CSA	Genital touching	Genital (both the abused and abuser had moles)	*	*	*F* = 0; *M* = 4
		Lachrymal		*	*F* = 0, *M* = 1
		Other			*F* = 0, *M* = 0
	CSA perpetrated by Buddhist ritual officiant (and monk)	Genital		*	*F* = 1, *M* = 0,
		Lachrymal		*	*F* = 1, *M* = 0
		Other			*F* = 0, *M* = 0
	Subtotal:				Genital: *F* = 1, *M* = 4 Lachrymal: *F* = 1, *M* = 4 Other: *F* = 0, *M* = 0
Total					Genital: *F* = 3, *M* = 26 Lachrymal: *F* = 9, *M* = 7 Other: *F* = 5, *M* = 1 Total: 51 (*F* = 17, *M* = 34)

*Note*. CSA = child sexual abuse; IPV = intimate partner violence.

Everyone who was recruited to the study agreed to participate and none dropped out at any point. Once we transcribed the audio and visual recordings and compiled fieldnotes, we analyzed the views of informants and examined their expressions to see how both meaning was derived from the context and attributions were made. We paid attention to the informants’ cultural registers and the use of popular Khmer culture references.

By taking as a starting point the Khmer terms as local concepts and idioms, we avoided the “category fallacy,” defined by Kleinman (Kleinman, 1987, p. 452) as “the reification of a nosological category developed for a particular cultural group that is then applied to members of another culture for whom it lacks coherence and its validity has not been established.” Khmer terms are spelled using Huffman et al.’s (1970) adaptation of the International Phonetic Alphabet transcription rather than transliteration, to help nonspeakers pronounce the terms easily and consistently. Words and expressions in Khmer characters are included.

## Results

### A Blighted Woman

One group of moles serves as an omen wherein a person (usually a woman) is blighted and brings misfortune to herself and her intimates. People designate this blighted state using the Khmer word /aʔpʰoap/, that is, the negative of /pʰoap/, a derivative of the Sanskrit and Pali root *bhavya* or *bhabba*. According to Buddhist dhamma, for example, Vin.i.17; SN.iii.27, being *abhabba* means being unfit and incapable, for example, of higher truths and salvation. In popular usage, it means that a person is incapable of achieving happiness for themselves and their intimate partners.

**Figure 1.**
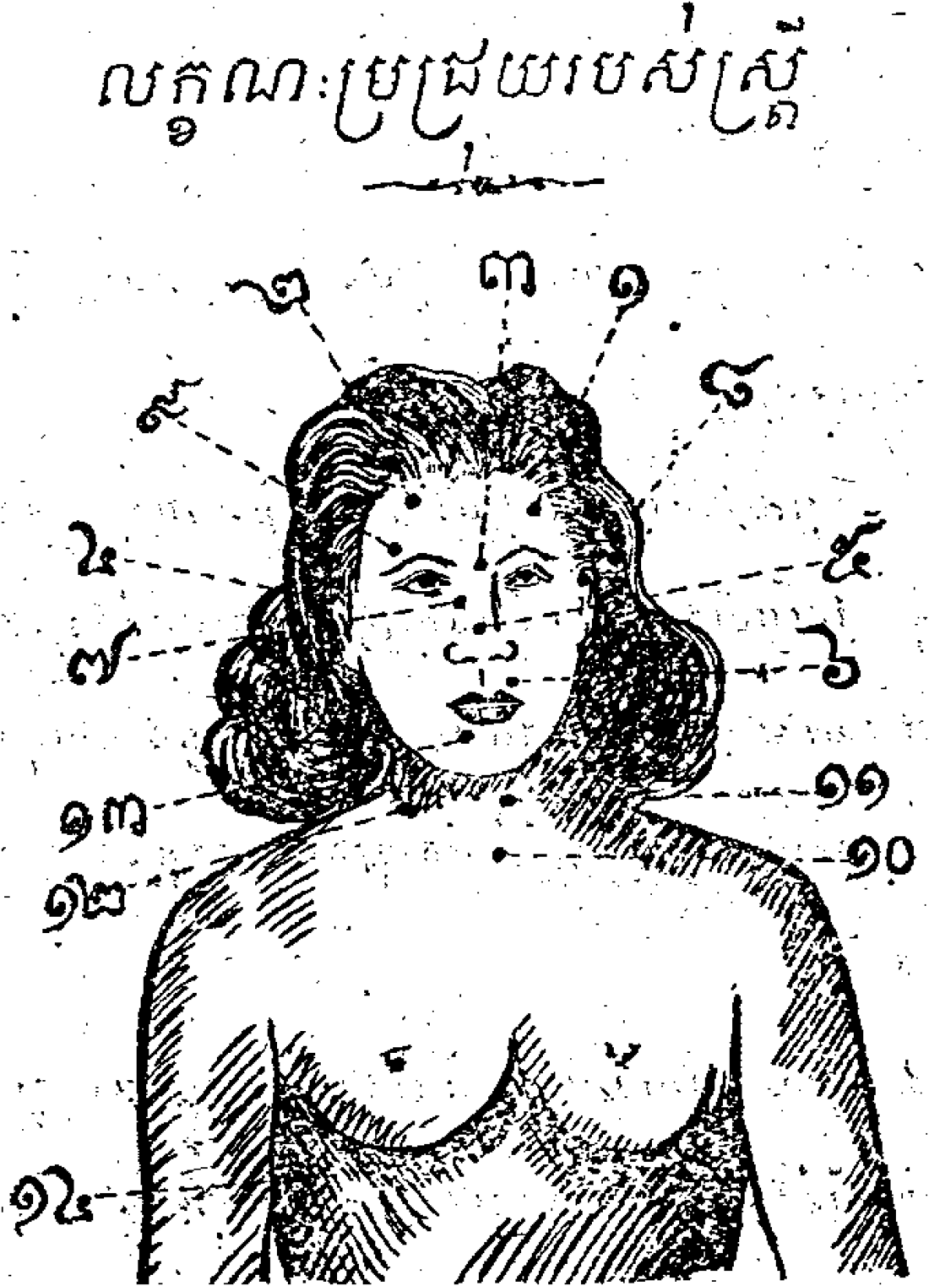
Distribution of moles (Nuon, 1966). The Khmer labels are the numbers referred to in Table 2.

People want to know whether a woman is /aʔpʰoap/, and the most obvious markers are moles, known literally as “incapable moles,” /prɑcruy ʔaʔpʰoap/ 
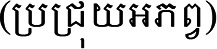
 and figuratively as “moles that signal an inauspicious consequence.” People consult astrologers or buy manuals that catalogue the links between the location of a mole and the prediction of relevant consequences. For example, “Pyiekɑɑ sastra 
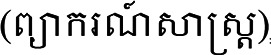
” (Prediction methodology) (Nuon, 1966) maps moles separately for men (pp. 48–50) and women (pp. 51–53). Figure 1 has been taken from this manual. It shows 15 different types of moles and their anticipated consequences.

**Table 2.** Moles on a Woman's Body, Presenting Only the Moles That Denote Bad Omens.

Original number	Location	Character	State of marital relations	Fate of child or children	Social relations
1	Left or right temple	Not sexually satisfied in marriage.	Constantly cuckolding husband.		
2	Forehead		Will break up within five months.	Their infant will die.	
3	Between the eyebrows	Unintelligent and incapable of independent living.		Infertility, stillbirths, or neonatal deaths.	Disliked and isolated.
5	Nose	Quick-tempered, self-righteous, and rigid.			Cannot accept responsibility and blames others.
6	Upper margin of the lips	Delinquent, looks for trouble, and pestering nature. Her behavior is akin to that of a prostitute.	Repeated breakups with multiple husbands.		Badmouths people and cannot fit into society.
7	On the nose bridge just below the right or left eye	Lacks virtuous character, brings misfortune on oneself and one's family, and is a spendthrift. Hot-tempered, quick to weep for no reason.			
9	Upper margin of the right or left eyebrow	/abhabba/. Self-obsessed, opinionated, jealous, and arrogant.			Alienates people by boasting, and denigrating and demeaning them.
10	Midline of the lower throat	High level of violent anger (*dosa*) and the heart of a robber.	If angered, she will be so violent that she may even murder her husband.	Can kill her child(ren).	Can murder her neighbors.
11	Left throat	/abhabba/.			If she acts kindly toward others, she will suffer bad consequences. It is better for her not to attempt to integrate with people.
13	Lower margin of the lips	/abhabba/, incapable of succeeding, and brings misfortune and destitution upon herself.			Efforts to get support from others invariably fail.

Table 2 shows how a mole signifying /aʔpʰoap/ is thought to predict consequences for a woman and her husband and children. Of the 15 types, 10 signify a blighted woman, and Moles 9, 11, and 13 are explicitly designated thus.

### Lachrymal “Mole That Is the Scaffold of Tears”

The lachrymal mole appears on either cheek, under the eye and is literally called “mole that is the scaffold of tears,” /prɑcruy rɔɔŋ tɨk pnɛɛk/ 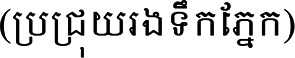. Table 1 includes other forms as well, such as the “mole that kills the husband” and its variant, “the mole that devours the husband.” Here, I present three vignettes, two involving tear moles, and one involving a mole that kills the husband.

#### A lifelong load of multiple crief—the case of ly sophoan

We met Ly Sophoan in 2018. When the Khmer Rouge came to power, she was aged 10 years. The Khmer Rouge evacuated the population to the countryside and took many children into the Youth Mobile Team, the /yuʔveaʔcʊən kɑɑŋ caʔlat/ 
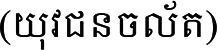
, literally, “a mobile military unit of juveniles” (Eisenbruch, 2007). Sophoan was forced to toil in the mobile team and witnessed the Khmer Rouge executing her father. This way, the young Sophoan set out on a path of lifelong tears and sorrow. Sophoan paid attention to a growing line of moles that extended from under her eye, down her cheek. She called this part of her life the first chapter of what was to come in her duty of “carrying a family load,” as though she were a porter carrying containers suspended from a carrying pole, an /ʔɑmraek/ 
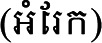
, across her shoulders. Sophoan summarized the rest of her life as a journey of carrying a series of extra loads. After liberation from the Khmer Rouge, she had to carry her emotionally broken mother. Her first marriage was to a drunkard who beat her. He had lots of children through liaisons with other women throughout their marriage. In her words, she shed “hot tears.” After she left him, Sophoan's load increased as she had to care for her sick children and her mother who was aged and disabled. Desperate to cut what she perceived as not only a marker, but also the cause of her misfortune, Sophoan tried to excise her “tear mole.” For five days, she repeatedly stabbed the area around the base of her mole using unsterilized sewing needles and dabbed it with soapy water mixed with red quicklime to kill the root of the mole. She was left with a white crater. For a while, she felt remoralized. Tragically, the excision did not end her tears. A while later, her two older sons were killed when their motorbike crashed.

Sophoan believed that her “tear mole” was a mark of her karmic destiny to be blighted /ʔaʔpʰoap/ 
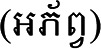
 and to bear an accumulation of containers on her carrying pole, that is, effectively, a lifetime of tears. Although the excision temporarily bolstered her morale, removing a marker cannot remove the root cause, and the karmic debt had not been discharged. She wished that she had consulted a monk to ritually excise the mole.

#### Child rape by a monk: The Case of Hanni

Hanni, a girl aged 5 years, was raped by a Buddhist monk in 2017 during the Kathen ceremony, which is the annual Buddhist festival celebrated at the end of the rainy season retreat. Hanni and her five siblings lived with their father, a tuktuk driver, and mother, a garment worker, in the poorest quarter of Phnom Penh. Her parents had always feared that she would face disaster. In a “conception dream” that heralded pregnancy, her mother walked along a creek when a cobra pursued her without spreading its hood. It coiled around one of her legs. Instead of striking at her, it sought her permission to live with her. When she asked why it would want to live with a human, it said that it wanted to be reborn as one. She promised to allow it to do so. Her husband did not want to have a child who had been a cobra in her previous life, and felt that the dream was a bad omen, connected with the Keng Kang Snake legend. Ignoring his plea for her to have an abortion, she felt that she had to keep her promise to the cobra.

Right after birth, they noticed a mark in the shape of the Khmer letter 
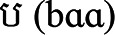
 between Hanni's eyebrows, which her parents and grandmother explained was the upside-down form of the letter 
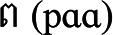
, which stood for the word /pɔpie/ 
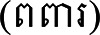
, the coiled portion of a cobra ready to strike. Even at the time of birth, she exhibited cobra-like characteristics, for example, she tried to bite the traditional birth attendant. There were three puncture marks on her buttock, which her parents assumed were scars from a trident that had fatally speared her when she was a cobra in her previous life. Hanni, the cobra, who was reborn as a human, carried the heritage of her former life. When she emerged as an aggressive and fearless little girl, her parents fretted. Her mother took her to see a healer who advised that as she had already been reborn in human form, she was safe from the dangers associated with her previous life as a cobra.

**Figure 2.**
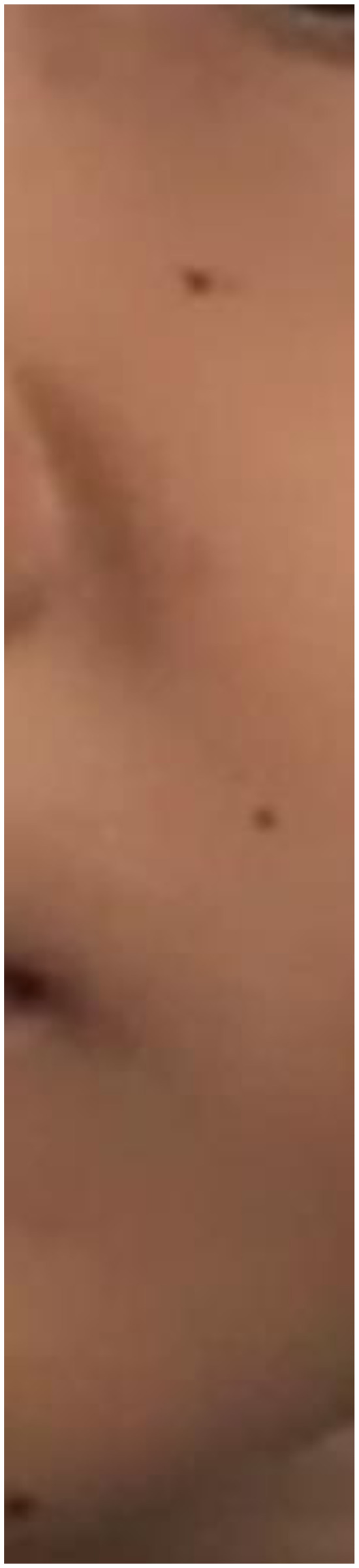
Three moles below Hanni's left eye, running across her lower cheek and chin. These omens foretold perils and sorrow.

**Figure 3.**
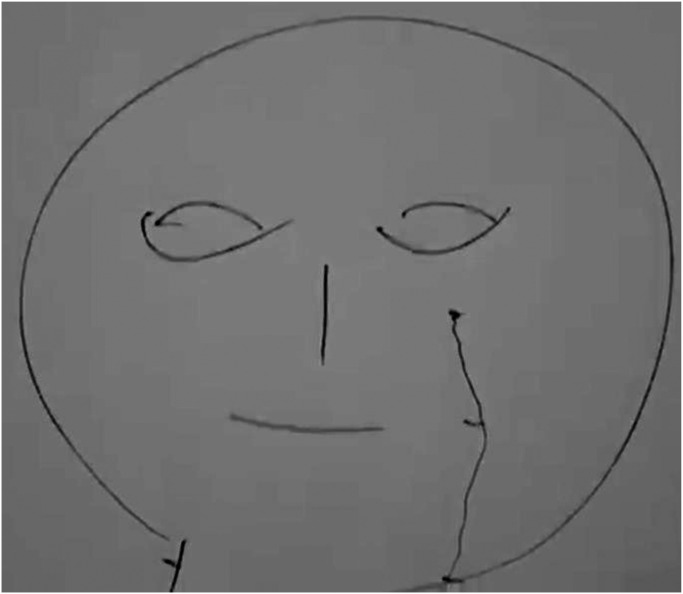
Sketch by Hanni's father of the tears flowing down her face, along her tear moles. These tears were predicted by the ominous moles.

Hanni had a “mole that supports tears,” /prɑcruy rɔɔŋ tɨk pnɛɛk/ 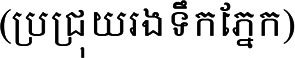 under her left eye. Two more moles developed on her left cheek, running along a line down to the chin. Hanni's father drew her face (Figures 2 and 3) and traced the trajectory of tears along the line of moles, representing the series of tragic mishaps she faced.

A day before the Kathen ceremony, Hanni's mother had a nightmare in which an ox ran in pursuit of her two daughters. While she managed to rescue her older daughter, the ox gored the younger one. An astrologer confirmed that the dream was an omen portending that Hanni would face peril. The next day, Hanni and her sister were at a temple with their parents for the Kathen ceremony. Among the monks, there was a 14-year-old novice who had been ordained just a few weeks earlier. He called the plucky Hanni to accompany him into a storeroom to get some soft drinks and there he raped her. Hanni's parents thought that the “tear mole” under her eye had signified many years of weeping ahead of her, indicating the sorrows she would face as a consequence of her karmic destiny that had been inscribed from her previous life as a cobra. Hanni's father, despite having discovered the local Christian evangelical church, was desperate to cut this ongoing misfortune. He hoped that he could scrimp enough money to pay the hospital to excise the three lachrymal moles, and believed that the flow of tears of misfortune would cease.

For Hanni's family, the web of causality came together. The rape was foretold as one in a chain of tragedies that she had already faced in her short life. Her parents could see the character of the hooded cobra in the fearless ways she had inherited from her previous life. These ways led her into danger in this lifetime. The “mole of misfortune,” along with his wife's dream of the ox and her calves the night before the Kathen ceremony, signaled that Hanni was doomed. One can see the karmic theory emerging in the image of tear moles. In her previous life as a cobra, Hanni had venomously bitten others. Now, it was her turn to face her karmic destiny.

#### Genital “Hidden Mole” That Lassos the Opposite Sex

Moles on the genitals of men or women are hidden from view in what people sometimes called the “heirloom, the personal secret, which one is shy to expose and should be kept hidden,” /kee kmaah/ 
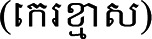
. If, unbeknown to women in the community, a man's mole is on the shaft of the penis, it is literally called, “the mole, the woman will pursue,” /prɑcruy srəy dəɲ/ 
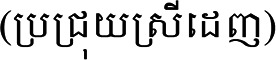
, and it will pull in crowds of women who seek to have sex with him, who is known accordingly as a man who “lassos women,” /cvak srəy/. There are also women who have hidden moles on their genitals, with which they are said to “lasso men,” /cvak proh/ 
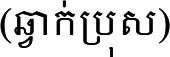
.

The lassoing is sometimes described in animal terms. A man with a birthmark on the tongue is likened to a bloodhound with its sense of smell. A man with a birthmark on his penis is likened to an elephant hunter. In each case, the penile mole draws the prey to the hunter. In an interesting sexual metaphor, Mr Ien, aged 73 years at the time of study, lived in Kampong Chhnang, had a penile mole, and said that penile moles ran in his family. Even if he was not sure who had one, he would suspect it whenever one of the men, for example, his uncle Dara, lassoed and lived with many women. The sexual power of the penile mole waxed and waned throughout their life cycles and was strongest among the young and middle-aged men. Mr Ien said that a purplish tongue radiated such potency that it would even affect a venomous snake, which would not dare to bite the person. It is the lasso, and not the man, that does the hunting. There are two possible reactions to the penile mole. Rithy blamed the omen as the cause for his bad conduct with women and his ultimate misery. Soriya did not blame the mole but welcomed it as a very useful weapon for potency.

#### Blaming the penile mole: the case of rithy

Rithy had been special from the beginning. He had a cockroach-colored tongue and two raised birthmarks on his penis, which made him particularly potent. He was born in a caul, which parents generally take to mean that the newborn has a spiritual master-teacher who would protect the child as long as its parents—and later, the child in its own right would make propitiatory offerings to the spiritual master throughout its lifetime. When I met him, Rithy was in his 30s and lived in Siem Reap. He had lassoed 15 women, who were mainly vulnerable widows and young adolescents. He and his mother said that these women had been uncontrollably drawn to him because of his penile mole. The 15 woman had a genital mole herself, which made the mutual attraction between both of them so powerful that she broke away from her marriage and family to run after him. Each time, the last-but-one was destroyed for having uprooted her life, sometimes abandoning a husband to be with Rithy. These women were despised by their communities for their shamelessness. Rithy had wrecked their lives. He had ruined his life, too. He was on the run from both vengeful members of his victims’ families and the law.

Looking back, Rithy blamed his victims for having caused him harm. His penile moles, he thought, should have given him a charmed life, at least in bed. However, each of these women who had been lassoed by his penile moles must have been of bad character and lacked virtue, /kʰaat leakkʰ/ 
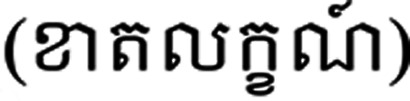
. Rithy espoused the popular view that such women bring ruin upon their husbands. He was broke and lived in a rented room in a violent neighborhood. He wondered why he had to philander, often with married women, by drawing them to himself like moths to light. He understood why he had destroyed himself in such a way by falling, on the “road to ruin,” which is the Khmer mode of depicting moral decay involving “women, alcohol, and gambling” (*apāyamuk*).

Rithy consulted a renowned monk-diviner, who performed an augury ritual. The monk asked him to mount a plank that rested on a grid of four blocks. He then announced the names of master-teachers who had been violated in succession—the master-teacher of the umbilical cord–caul, teacher from birth, and so on. The augury pointed out the “wrong to the master-teacher of the caul–umbilical cord,” which was just what his mother had recalled from his birth. Having confirmed the diagnosis, the monk told Rithy to make regular offerings to the master-teacher of his caul–umbilical cord. The monk handed over an effigy he had made of Rithy as a newborn. It featured the conical red “hat” that represented the caul on a ripe coconut wrapped in shiny green foil, which symbolized his body. He enveloped the coconut in raw cotton, which he slung diagonally from top left to bottom right, representing the cord that had been draped across him at birth. Rithy returned home and placed this assemblage in the center of his platform. Within a matter of weeks, Rithy's business picked up, and women stopped flocking to him.

Rithy realized that even though the magical power of his penile mole had been quelled, there was more to it. The monk helped him see that from early on, he had failed to respect the moral code, starting with that toward his spiritual master-teacher, which had led to his *apāyamuk,* that is, his carousing in casinos on the Thai border, where his wallet was emptied. The monk helped him see that he had become morally bankrupt. The families of several wronged women were in hot pursuit, intent on avenging the hurt he had caused their women and hoping to murder him. The police, too, were after him. Rithy had to move from town to town. Finally, he saw that his penile mole was an omen of misery but that he, and not his mole, had created his downfall. To cleanse himself of his bad karmic destiny and sever the significance of his penile mole as a continuing omen of misfortune and violence, Rithy became a monk. His story shows how a perpetrator may use his mole as an excuse to shrug off accountability, an act that monks describe as shameless impunity, or /ʔaʔlaccii/ 
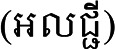
, a term derived from the Pali *alaji*, as though the mole is “not the self.” Rithy's brother-monks confronted him, and then symbolically rebooted his violated relationship with his master-teacher, thereby, it was believed, persuading them to restore their protection and blunt the omen of the penile mole.

#### The penile mole that brought misfortune on both spouses: the case of soriya

Soriya, aged 28 years at the time of this study, was a motor taxi driver in Kampong Cham. He attracted Sophoan, who was 10 years his senior, who quickly moved in with him, only to discover that Soriya's penis had no less than seven moles on it. In her words, he was a “hunter of women,” /prien nierii/ 
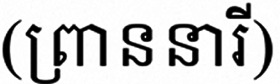
 in the sense of snaring succulent young flesh to enjoy. From the beginning, Sophoan had to literally share her bed with a stream of young women. Inexplicably, she ignored his sex acts with all these women in her presence. Over the next 30 years, Soriya had had at least 25 sexual relationships, including some with Karaoke girls, many of whom are indirect commercial sex workers. Sophoan was suspicious that some of these rivals had also played a part in the “lasso” by casting their magical charms to hook Soriya, as she metaphorically said, “to keep the hunter's eyes fixed on trapping their flesh.” A monk-medium went into a trance and told Sophoan that one of the Karaoke girls had bewitched Soriya by adding her menstrual blood to his food. Once, he had brought two young girls into their bed, and she just lay in the corner under the mosquito net and let him have his way with them. Brazenly, he told Sophoan that he needed these younger women as his “spare wheels.”

Sophoan desperately sought to rekindle his sexual appetite for her. The neighbors warned Soriya, as a married man with children, not to play around with other men's wives and that he would be better off “visiting the place (the brothel), where he could pay for the cakes he desired.” However, his penile mole did all the work for him. The women kept coming to him. The most recent (at the time of study) was Nari, who was nearly 30 years younger than his wife. She was a garment worker who rented a place near her factory in Phnom Penh.

Sophoan consulted a love-charm specialist healer to cast a spell on Soriya, and he prepared and activated two sets of Yantra. The first was a “love charm to smash up” /snae bɑmbaek/ 
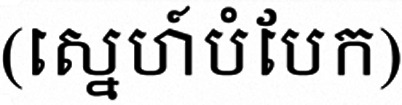
 the sexual chemistry between Soriya and Nari. He gave Sophoan 20 copies of the Yantra, on which he had inscribed Nari and Soriya's names. Sophoan had to first pulverize the Yantra by treading on it with her heel while reciting a Pali stanza, and then incinerate the pieces. It was as if she was literally smashing the bonds among her, Soriya, and Nari. The healer gave Sophoan a second Yantra called “love charm to put back the broken pieces” /bɑŋruəp bɑŋruəm/ 
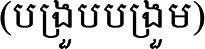
. She had to throw the Yantra into the fireplace in the kitchen to induce her husband to feel nostalgia for her “rice pot” and return to her. Then, she had to take the copper squares on which the healer had engraved another Yantra and bury them in the ground at the approach to their house, where the Yantra inscriptions would inform the deity Dharani to soften Soriya's heart and convert his hostility toward her and their children into love as soon as he stepped on the earth. A third Yantra was a diagram of Sophoan and Soriya facing each other in reconciliation. Sophoan tucked this discretely into Soriya's pillowcase. In the fourth Yantra, Sophoan had to submerge the inscribed copper squares in water for a while, invoking the help of Ganga, the Hindu goddess of the river Ganges, known in Khmer as /preah mae kʊəŋkie/ 
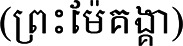
, here, a metaphor for cooling off the heated and fractious relationship. She had to take the copper squares out and give the magical water to Soriya to drink. Within days, the love charms worked and her husband abandoned Nari and returned home as his old self. However, over the course of a year, the power of the penile mole took hold again.

On their son's wedding day, Sophoan invited Soriya, who fulfilled his obligation as a father by participating in the ceremony. Later, when Sophoan cajoled him to stay, Soriya went berserk and tried to slash her with a knife. To defend herself, she threw battery acid at him. It splashed onto his face, leaving him with third-degree burns that severely disfigured him. Fearing arrest, Sophoan fled. However, she could not give up on him. Soriya forgave her for the acid attack, but when she visited her husband in hospital, he was busy having sex with Nari in the hospital bed. That was the last straw. Sophoan filed for divorce. Two years later, she stated that the problem was not the love charms cast on him by various women from time to time, but rather his penile moles, as he had been born with them and would die with them, which meant that he would never change his character. She remained unimpressed by the words of the traditional healer she had consulted that even a penile mole could lose its power if the man was determined to embrace Buddhist virtue and moral conduct. Soriya never severed his connections with his penile mole. Finally, he suffered the karmic consequences of his behavior, as he was permanently disfigured by the acid attack. The penile mole was the marker of what could never change. Taking the metaphor of the hunter of human flesh a step further, traditional healers tell their male patients that after receiving magical ritual treatment, they are prohibited from consuming the “flesh of a biped,” a /sat tviʔbɑt/ 
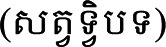
 or /sat cəəŋ pii/ 
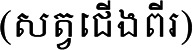
. The biped here refers to a woman outside marriage, and the language “two-legged” has the connotation of a dishonorable woman who, one may say, has “open legs” for such men. Sophoan meant to say that Soriya was a hunter of prohibited but irresistibly tasty and cheap women. For her, the hunter and his prey were each of a bestial character.

#### Even a monk commits CSA with his penile mole: the case of buntha

In the case known in popular media as “The Bizarre Love Triangle Involving Two Monks” (Anonymous, 2018), one drunken night, Venerable Som Kimsan, the chief monk of Kirivong Sleng temple in Samrong Tong district, Kampong Speu province, and his long-time drinking companion, deputy chief monk, Seng Buntha, seduced an orphaned temple servant. After some months, her pregnancy began to show. In a drunken quarrel over the child's paternity, Som Kimsan pulled out a machete and slashed his deputy's arms and legs. The two were defrocked for having violated the monastic code against sex. Within a few days, we followed up at the temple and then again a year later, and spoke with two Buddhist ritual officiants. We met Seng Buntha's father, who had his own explanation for the role of his son's penile mole in his downfall. Buntha's first marriage was a disaster, his second marriage to an Australian-Khmer woman ended in divorce, and he entered monkhood in pursuit of solace. Buntha's father believed that this misfortune was because his son's penile mole had “lassoed women,” but he swore that his son had never committed the monastic offense of *pārājika* by violating the first rule of the Vinaya code and having sex with a woman, let alone an underage girl.

#### The mole that devours the husband

There are additional variations in which “the mole that kills the husband,” /prɑcruy sɑmlap pdəy/
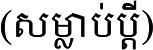
 or “the mole that devours the husband” /sii pdəy/ 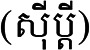 is seen as dangerous or lethal to a man (Table 1). A murderously angry woman who can kill her husband, children, and neighbors, is marked by a mole on the midline of the throat—the location alluding to the act of “wringing the necks of her victims.” A woman with a mole symbolizing peril cannot retain her husbands as they die one after another. Mr Ien told us about Rachada, a woman who was in her late 40s who lived near his daughter's house. Although she was of good character and had never been with other men, people noticed that Rachada had a dark, purplish tongue, which was perhaps a nevus. Her newly married husband had died mysteriously soon after their wedding. She remarried a Buddhist ritual officiant. However, he died, too. The third time around, she moved in with another ritual officiant who specialized in arranging cremations. Mr Ien remarked that he was still alive, which must have meant that given his everyday work of handling corpses at the crematorium, he must have acquired some special powers to cancel out the lethal force of her mole. I came across this type of mole in the early 1990s, when I met several traditional healers. Kruu Lonh and I discussed his palm leaf manuscripts, including the section on the “mole that devours the husband.” He said that men should avoid marrying such women. Even if such a woman was to marry 10 men, each of them would die.

### Social Media

**Figure 4.**
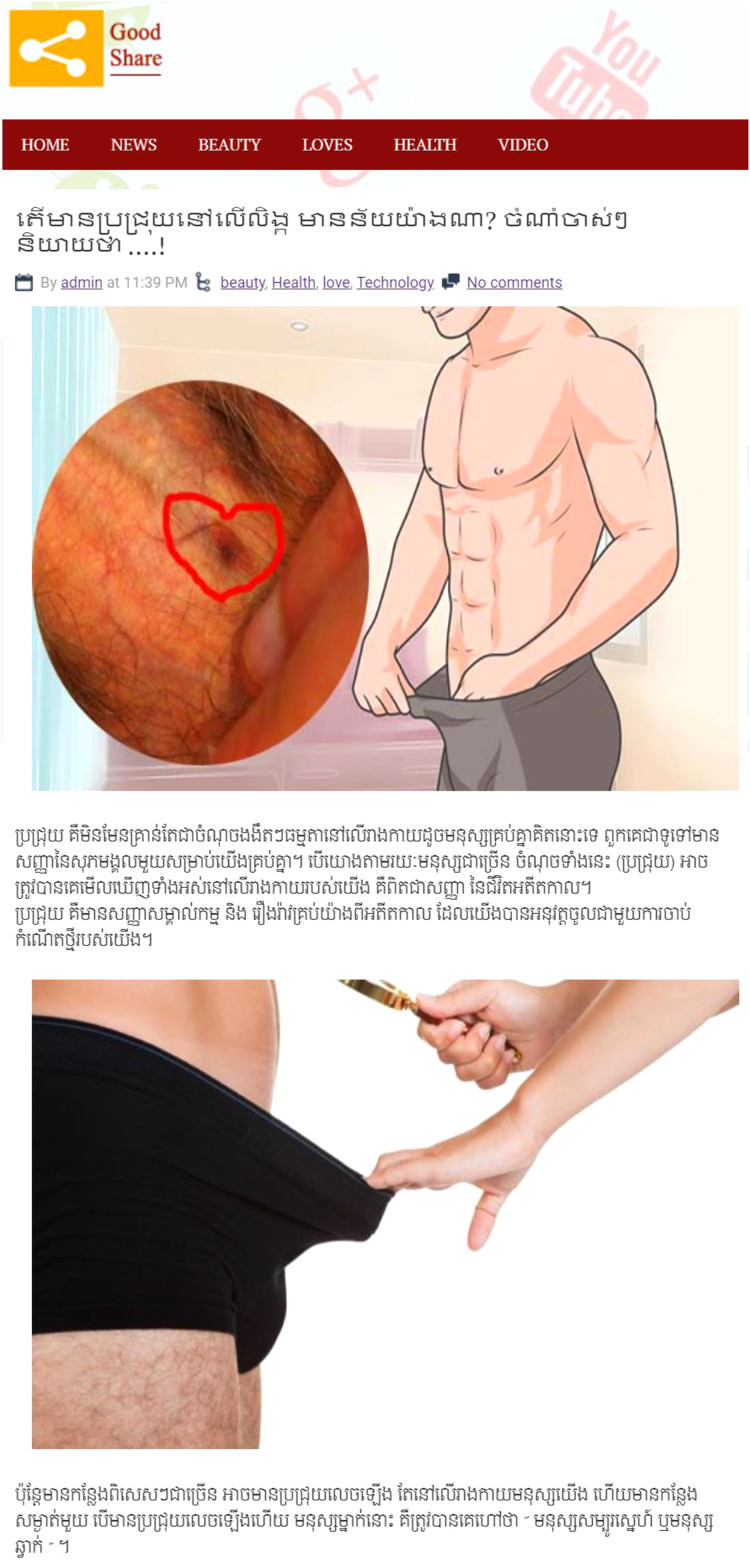
A popular blog showing details of a mole on the penis.

Ideas on the sexual magnetism of the penile mole remain popular in the Khmer-language media targeting both men and women (see Figure 4).

Portrayals of and stories on penile moles in the media target young women who are interested in beauty, love, and family-making. According to the Kanha website, if the mole is on the side of the penis, their lust will be confined to one partner at a time. If it is on the glans or the underside of the shaft, the man will be uninhibited in bed. If it is on the base of the penis, his tendency to lasso a large number of women will start from a young age and remain this way well into old age (Anonymous, 2017).

Another popular website, Piphup Kamsan, has articles on solving problems with health and sexuality (Anonymous, n.d.). In an interview, Roath, a famous female fortune teller on the foreshore of the Royal Palace, called the penis a “secret weapon” that is hidden from public view until a woman comes within range, and explained that when it becomes erect like a barometer, she is drawn in as if struck by lightning. Even if the man is not handsome or sweet talking, women cannot escape being lassoed by his considerable “magical love charm.” All it takes is the blink of an eye. There is no need to resort to a love charm: It is built in from birth. She tells these clients that it is their destiny to be unfaithful, without any regard for the well-being of the wife and children, with hordes of women running after them. Her clients complain and ask why they lasso women but never money. Some beseech her to get rid of the mole's lassoing effect, and she claims that she can help by performing a substitution ritual in which she symbolically excises the mole.

## Discussion

The penile mole is a cultural idiom of coercive control through a power imbalance over vulnerable women. Coercive control is described in Cambodian society (Eisenbruch, 2018c; Hilton et al., 2008; Salan, 2005) as follows. If a man exploits multiple women who seem to have lost control over their faculties and are drawn magically to the mole on his penis, he engages in a form of coercive sex. This is similar to phenomena that have been reported in other societies. For example, van der Watt and Kruger (2020) described the abuse of traditional juju ceremonies in the Democratic Republic of Congo to induce obedience to a trafficker and prevent the target from absconding. Similarly, anthropologist and psychotherapist Taliani (2012, p. 582) described the “multiple layers of domination,” such as voodoo or juju, over Nigerian immigrant women in Italy, which Baarda (2016) depicted as a coercive mechanism. Culturally mediated magical forces place a victim under the spell of a perpetrator who can deny any conscious intention or action on his part to “lasso the woman.” Sharp (2014, p. 1408) spoke of “religious coercive control” by which, at least in Christian settings, “abusive husbands use religious beliefs, values and doctrine as a means to coercively control their intimate partners.” A man using the perceived power of his penis to “lasso” a series of women who acquiesce to years of chronic emotional abuse effectively exploits a form of “coercive control” by relying on culture. Chen and Triratpan (2020) conducted a fascinating analysis of judgments in Thailand, which is culturally similar to Cambodia, to check whether a perpetrator of sexual assault was guilty of a criminal offense during a religious healing ritual, that is, whether he had engaged in “fraudulent sex” in the context of “religious fraud.” They found that the Penal Code had traditionally focused on the stupidity or ignorance of the female victim in a paternalistic manner, excluding religious fraud as constituting sexual assault, whereas the newer view that had emerged was that a victim is incapable of resisting. In the case of a penile mole as demonstrated here, the informants are placed in a rather similar position in line with the older Thai way of thinking. The man who exploits his male hegemony hides behind the mole to maintain his impunity. A tiny handful of monks and traditional healers have misled their patients by claiming that sexual assault is a part of the treatment they provide, but the overwhelming majority of monks in Cambodia preach nonviolence, including in intimate relationships. This fact is consistent with work by Kanukollu and Epstein-Ngo (2015) in Bhutan, where Buddhist monks were aghast that women should think their menfolk had the right to abuse them, a view strongly out of alignment with Buddhist teachings.

Scientist–historian Kantha (2016) classified Shakespeare's use of “naughty” synonyms for the penis, as in “Ensign Pist” in Henry IV, “potent regiment” in Antony and Cleopatra, and “naked weapon” in Romeo and Juliet, where mate attraction is equated to warfare from the man's perspective, involving the capture of a woman. In a lecture at the Royal College of Surgeons, Bett (1952) reminded his audience of a man who inserted an artificial bone into his penis, unwittingly emulating the “os priapi” of squirrels, otters, walruses, and whales. In her groundbreaking work titled *Sexual Politics*, Millett (1970) demonstrated how, in a patriarchal society, such as in Melanesian men's houses, the penis is used as a weapon of male power, sexuality, and violence. Javaid (2018), in her feminist study of hate crimes, here homophobic violence, reported that the penis is used as a weapon of power over the victim. Mullins (2009) noted the cultural factors prevalent in the perpetration of sexual violence during the Rwandan genocide in 1994, where survivors saw the penis as a perpetrator's weapon.

In the Indianized states of South and Southeast Asia, including Cambodia, the *liṅga* is a stone phallic symbol and serves as an emblem for the worship of Shiva. The Linga Purana of Hindu religious texts explains that everything is issued from the *liṅga* and that the one who possesses it is the Supreme Being (Cormier & Jones, 2015). The *liṅga* is a portrayal of masculine potency and charisma, and the penile mole makes it especially so.

There is a social and economic context at play. Men in contemporary Cambodia may feel threatened by the loss of their leadership in the family. In societies where they traditionally enjoy hegemonic masculinity, men who are thwarted of their power can become hypermasculine and may, for example, acquire women as tokens “in order to reestablish lost hegemony” (Banwell, 2014, p. 53). The symbol of the penis in Cambodia as an instrument of power over women is a fascinating illustration of hegemonic masculinity, which supports Hooper's (2001) argument that, in a phallocentric discourse, the penis and therefore masculinity are positively valued culturally and imbued with power.

There are parallels between these Cambodian observations and those reported by Cameron (1992) in her study of male American college students in their lexicon for the penis. Whereas the Cambodian material has centered on the powerful Linga of Shiva, American students have metaphorized the penis as an authority figure, wherein in some metonymic sense, it is the man, his “rod of lordship” through whose symbolic power he himself rules. The Cambodian material shows the penis, through its mole, as a magical weapon that makes a woman fall for a man by lassoing her. The American students collocated “love” and “war” terms as metaphorical linkages between sex and violence, in relation to cultural norms of masculinity.

It is striking that the Cambodian men who seemed so powerful in brandishing their penile moles, at least to themselves, often led disempowered and downtrodden lives, although not to the same extent as the women they lassoed. I am reminded of the insights of psychotherapist Berke (1989, p. 425) that the phallus of a man may not measure up to the weight of his own ambitions or inner doubts, to which one possible reaction is that the phallus becomes “an all-powerful weapon that can grant every wish, destroy every danger and vanquish every pain.”

In this article, I shed light on a cultural perspective of coercive sex, in which a perpetrator to some extent blames his penile mole rather than himself for controlling women. When the man says it was not him, it was his penile mole, it is reminiscent of the depiction by Potts (2001) of “the man with two brains,” in which hegemonic masculinity is exteriorized, the penis coming to represent the man, “with its own will outside the man's conscious control.” The depiction of the Cambodian penile mole as a metaphoric “lasso” of women, one over which the man has no control, could be interpreted as “penis primacy” (Adeniyi, 2022) and as an alibi for toxic masculinity (Cameron, 2006).

The belief system described invites us to consider that the women have reduced free will before they are lassoed into abusive relationships from which escape seems impossible for far too long. These are the hallmarks of coercive control, which is at stake for so many of these men and women in settings of wretched powerlessness, sometimes poverty. Based on the six cases, a more complex causal relationship emerges where both the perpetrators but also the victims, as well as the wider community/society seek, to explain certain behaviors as being conditional on what are believed to be the forces beyond their control.

Moles are cultural metaphors. They are markers on the skin, which Yadlin-Gadot and Hadar (2018, p. 281) described as “a metaphorical surface inscribed with social and cultural meanings, and imbued and marked by psychic significance.” The name of the lachrymal mole is /prɑcruy rɔɔŋ tɨk pnɛɛk/ 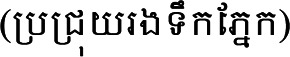, which glosses as “the mole + bears, supports, scaffolds + water-eye.” As demonstrated in the cases of Sophoan and Hanni, the tear mole is an orientational metaphor, a predictive marker of the rivulets of tears the woman is destined to weep in response to successive waves of misfortune. They are truly “women of misfortune,” or /srey ʔaʔpʰoap/ 
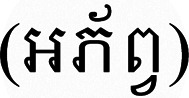
, which is a word derived from the Pali *abhabba*, which means “unable.”

The penile mole is known by various names, such as /prɑcruy lɨŋ/ 
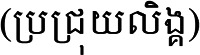
, which glosses as “the mole + penis.” In mythological terms, it refers to the mole of the *liṅgam*, that is, Hindu deity Shiva's phallus, and is a symbolic metaphor, a magical source of sexual energy, and the creator of new life. It is also known as a “secret weapon,” /ʔaavut sɑmŋat/ 
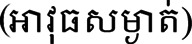
, a “stealth mole,” /prɑcruy sɑmŋat/, 
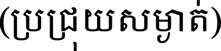
, and in the metonym, the “secret weapon” is concealed in the private parts and magically lassos its target and satisfies the owner's lust. Rithy's weapon was affected by his relationship with the master-teacher of his umbilical cord. Soriya experienced a balance of forces between his penile mole and a counterforce that came from his love triangle involving two women who competed in “hunting the hunter.” Finally, the power of Buntha's penile mole was potent enough to triumph over his obligation to the Vinaya code as a monk.

Cambodia is an “Indianized State,” and the Cambodian construction of birthmarks as omens echoes the mechanism of “human marks” found in ancient Indian medical systems. Zysk (2015) documented the literary category of the Sanskrit *Jyotiḥśāstra* text, which deals with astrology and omens and codifies two separate collections of verses, namely the marks of men (*puruṣalakṣaṇa*) and women (*strīlakṣaṇa*). These verses make predictions based on the examination of moles (*tilaka*) on the skin. There is a remarkable similarity with the Cambodian divination of moles as detailed in the traditional healers’ palm leaf manuscripts and printed manuals (Nuon, 1966), which I have collected over several decades. Some of these include the codification of moles of men and women; the diagnostic process up or down the body; the binary system, with contrasting positive and negative values between the left and right sides of the body; and conditional sentences with protasis and apodosis, as in “if …, then …” with the position of the mole being seen as an omen predicting a specific fate such as sickness or sudden death.

The case of Hanni, reborn into this life bearing marks from a previous one, is no surprise in Buddhist society, and is a notion that gained respectability in a recent commentary by Egger et al. (2019), in *Dermatology*. The belief that birthmarks occur when the mother has a strange experience during pregnancy, as was true of Hanni's mother, has been called “material impression.” Stevenson (1993, 2001) studied Burmese informants who recalled the lives of people who had died of venomous snakes. He found that birthmarks were often located where therapeutic incisions were made at the site of such snakebites. Keil (1991) reported similar findings in Myanmar, Thailand, and Turkey. These presumed links with previous incarnations are considered omens of things to come, for example, in Thailand (Tucker & Jürgen Keil, 2002) and northern India (Pasricha, 1998). In a karmic sense, a mark of a previous life is also a mark of the future.

Genital moles are “secret moles,” /pracruy leak kaa/ 
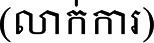
, and the prospective female partner will have no warning until it is too late. Villagers know the expression “dog and woman lassoed into one another,” /cvak nɨŋ srǝy/ 
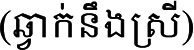
, where a series of vulnerable virgins, widows, and married women, become receptive to a man with a penile mole. The second stage in the control is that this man would engage in rough sex, like a dog, leading sooner or later to pregnancies. Finally, this man would lasso the next victim (another round of emotional abuse) and abandon his partner and their brood. He is, as depicted in a popular expression, “a shitting dog leaving its shit lying there without covering it up,” /cuh caol/ 
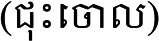
.

Another cultural motif of birthmarks and moles is that they can be “hidden.” Jaberg (1957) described this in Old French and Romance scholarship, for example, in Boccaccio's story “Zinevra, Bernabò e Ambrogiuolo” in the Decamerone. In the findings reported in this article, a real mole is patently obvious on the face or the arms, whereas a “shy” mole hidden on the male or female genitals is more elusive and is thought to cause coercive control. This mole hides in “the shy and ashamed place,” as reflected in the compound word /kɑnlaeŋ kee kmaah/ 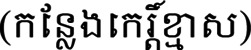, with three elements, “the place,” /kɑnlaeŋ/, “the reputation or honor,” /kee/ 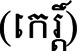, and “ashamed, or genitals” /kmaah/. The revealing element in this compound word is /kee/, which is derived from the Sanskrit word *Kīrti*, which connotes royal reputation (Cecil & Bisschop, 2019).

The findings on a Khmer cultural reading of hegemonic masculinity and coercive control do not necessarily apply to all or even most men and women in Cambodia, where traditional beliefs may be evaporating under western influence. Efforts have been underway around the world, including in Cambodia, for “transformative masculinity,” in which patriarchal structures are made visible and men are engaged in the fight for justice (Diakonia, n.d.). A Cambodian study by My (2022), a Khmer researcher, demonstrates the emergence, at last in urban Phnom Penh, of “caring masculinities.” Nevertheless, I argue that the findings capture the reality of rural people and provide insights into a local logic obscured from the view of those involved in programs aiming to reduce IPV and coercive control, in which, as Lilja (2012) describes, “universal” versus “particular” representations of nonviolent masculinity are fostered.

## Conclusion

Curious beliefs around penile moles have been reinterpreted as a far more sinister set of beliefs that are used to justify multiple acts of coercive control over women in the lives of several couples. The penile mole in rural Cambodia is an omen of magical power. It is thought to have magical charisma to lasso several financially, socially, and emotionally vulnerable widows, divorcees, and single mothers, who tend to be trapped in harm's way. Rithy's case sets the scene with a typical story of a man whose penile mole is considered responsible for his act of drawing in dozens of vulnerable women and illustrates how the mole ultimately wrecked not only the lives of the women he lassoed, but also his own. There was evidence that the monk successfully dissuaded Rithy from continuing his cycle of abuse and that this intervention was so persuasive that Rithy chose to enter monkhood in his own right. Soriya's case shows the pernicious consequences in which a man in a “love triangle,” said to have completely lost his judgment as a result of his penile mole, ended up becoming a victim of an acid attack at the hands of his enraged wife. The story of a chief monk whose mole is seen to draw a girl into sex and pregnancy with him illustrates that even Buddhist monks are ordinary men and not exempt. A mole, unlike other omens I have documented in Cambodia, such as the snapping of a tree or the flight of an ominous bird (all of which are singular events and are simple, conditional “if this, then that” statements), is a “distal omen,” in that it is evident long before the event takes place. We may consider the birthmark a part of an assemblage of omens, such as Hanni's tear moles along with her mother's *nimitta*, her dreams, and Rithy's penile mole along with his infraction to his spiritual master-teacher. This way, an omen is an important marker of possibility or likelihood, rather than certainty. The findings show that the local cultural constructions reinforce coercive control and emotional violence in intimate relationships, and may sit uncomfortably with western rights-based approaches to GBV.

While it is not suggested that the cultural attributions reported in this article apply to all or even most Cambodians, social media and other sources have shown that the ethnographic findings reported in this article are real and retain some salience even in contemporary Cambodian popular culture. Monks, in promulgating the dhamma teaching, provide a powerful antidote to the popular beliefs described in this article. They show how it is not acceptable in Buddhism to blame IPV of any kind, including coercive control, on magical forces like penile moles. This way, monks can make a valuable contribution toward shaping culturally responsive mechanisms for intervention in the aftermath of an incident and to education and community awareness for the prevention of GBV.
